# The Neural Baroreflex Pathway in Subjects With Metabolic Syndrome: A Sub-Study of the Paris Prospective Study III

**DOI:** 10.1097/MD.0000000000002472

**Published:** 2016-01-15

**Authors:** Luca Zanoli, Jean-Philippe Empana, Nicolas Estrugo, Guillaume Escriou, Hakim Ketthab, Jean-Francois Pruny, Pietro Castellino, Dominique Laude, Frederique Thomas, Bruno Pannier, Xavier Jouven, Pierre Boutouyrie, Stephane Laurent

**Affiliations:** From the Sorbonne Paris Cité, Faculté de Médecine, Université Paris Descartes, Paris, France (LZ, J-PE, NE, GE, J-FP, DL, XJ, PB, SL); Department of Internal Medicine, University of Catania, Catania, Italy (LZ, PC); Assistance Publique–Hôpitaux de Paris, Hôpital Européen Georges Pompidou (LZ, XJ, PB, SL); INSERM U970, Department of Pharmacology (LZ, HK, J-FP, BP, PB, SL); INSERM U970, Cardiovascular Epidemiology and Sudden Cardiac Death (J-PE, XJ); and Institut de Prévention Cardiovasculaire, Paris, France (FT, BP).

## Abstract

The mechanisms that link metabolic syndrome (MetS) to increased cardiovascular risk are incompletely understood. We examined whether MetS is associated with the neural baroreflex pathway (NBP) and whether any such associations are independent of blood pressure values.

This study involved the cross-sectional analysis of data on 2835 subjects aged 50 to 75 years from the Paris Prospective Study 3. The prevalence of MetS was defined according to the American Heart Association/National Heart Blood and Lung Institute definition. NBP values were calculated from the fluctuation of the common carotid distension rate and heart rate using fast Fourier transformation and cross-spectral analysis.

The prevalence of MetS was 20.1% in men and 10.4% in women. Compared with controls, subjects with MetS (≥3 components), and those at risk for MetS (1–2 components) had lower NBP (−5.3% and −2.3%, respectively) and higher carotid stiffness (+13.5% and +6.8%, respectively). The negative association between MetS components and NBP was confirmed, even after adjustment for age, sex, and carotid stiffness. After stratification for blood pressure (BP) levels, NBP was reduced only in MetS subjects and those at risk with high BP. The NBP was positively associated with carotid stiffness in controls and subjects at risk for MetS. This association was lost in subjects with MetS, regardless of BP levels.

Subjects with MetS had reduced NBP values. The role of BP is fundamental in the reduction of NBP. The mechanisms that link carotid stiffness and NBP are inactive in subjects with MetS, independent of BP levels.

## INTRODUCTION

The study of the function of the baroreflex is of clinical relevance, as suggested by studies that show that autonomic dysfunction may play an adverse role in several cardiovascular diseases^1,2^ and that interventions that improve baroreflex sensitivity (BRS), such as physical training or β-adrenergic receptor blockade, may also beneficially influence a patient's prognosis.^3,4^

Autonomic dysfunction has been proposed as a causal link between unhealthy lifestyles, such as overeating and sedentariness, and metabolic abnormalities, such as metabolic syndrome (MetS).^5^ In addition, MetS is currently considered to confer an increased risk of type 2 diabetes and cardiovascular events, which is attributable only in part to the individual risk factors that concur in defining it.^6^ Therefore, it has been suggested that some of the excess risk detected in MetS should be attributed to a cluster of other factors associated with it. One of these factors could be the depressed neural baroreflex pathway (NBP), as suggested by the presence of sympathetic hyperactivity^7^ and depressed *global* BRS^8^ in subjects with MetS, particularly if hypertension is present.

**FIGURE 1:**
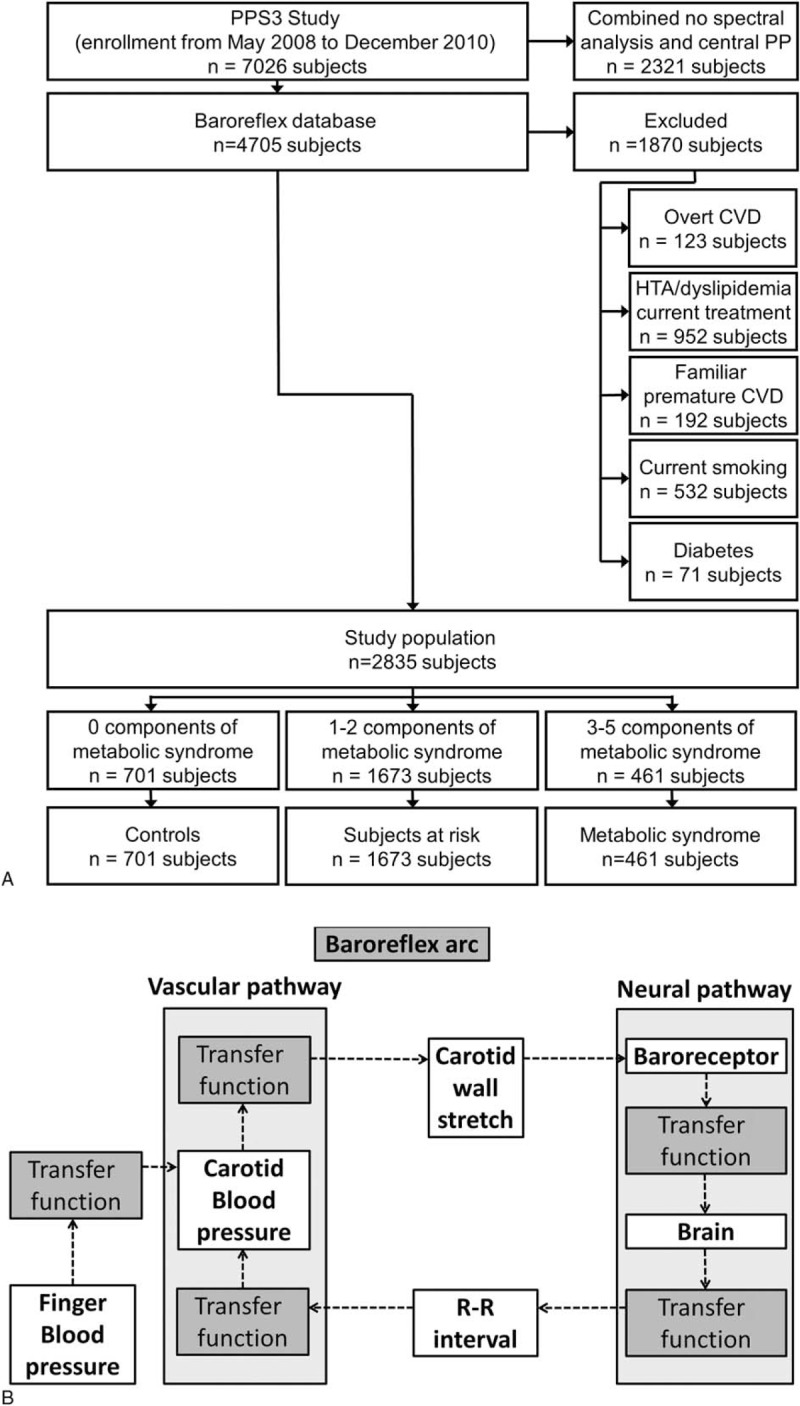
Panel A: flowchart describing the selection and categorization of subjects for the present analysis. Panel B: baroreflex arc with the vascular component of the baroreflex and the neural baroreflex pathway. CVD = cardiovascular disease; HTA = hypertension; PP = pulse pressure; PPS3 = Paris prospective study III.

Classically, fluctuations in BP are used to assess the *global* BRS, which is the result of both *vascular* (dependent to the arterial stiffness) and *neural* components of the baroreflex (the NBP) (Figure 1, Panel B). However, baroreceptors respond to deformation and not to pressure per se.^9^ Therefore, peripheral changes in BP might not accurately reflect changes in carotid bulb distension in subjects with increased arterial stiffness, making *global* BRS a poor indicator of the NBP. Moreover, both vascular components of the baroreflex and NBP can be jointly or singularly altered in several pathological conditions. Greater vascular stiffness depresses the autonomic regulation of the baroreflex in hypertensive patients.^10^ Therefore, considering that BP values in the high normal range represent 1 of the 5 components that lead to the identification of MetS, it is unsurprising that the vascular component (ie, carotid stiffness) may be altered in these subjects.^11^ Alternatively, in the absence of structural changes, autonomic dysfunction reduces neural transduction and dampens the responses of the baroreflex to decreases in BP in diabetic patients.^12^ In light of these findings, the role of the NBP in subjects with increased carotid stiffness and those at high risk for diabetes (ie, those with metabolic syndrome)^6,11^ should be clarified.

Recently, the study of the neural component of the baroreflex after fully controlling for the vascular component, deriving the NBP from carotid distension fluctuations^13^ (Figure 1, Panel B), has been proposed. To our knowledge, no studies have been carried out to study the NBP in subjects with MetS and to test the association between NBP and carotid stiffness.

We hypothesized that the effect of MetS on NBP is greater than the sum of the effects of each component of the syndrome. Therefore, we took advantage of 3 factors—a large cohort,^14^ the gold standard method for measuring arterial parameters,^15^ and the integration of validated tools^16^ in semiautomated processing—in order to study the spontaneous NBP and its association with carotid stiffness in patients at risk for diabetes.

## MATERIALS AND METHODS

### Population Studied

Cross-sectional Study: starting from the first 4705 persons with available baseline carotid echotracking measurements enrolled from May 2008 to December 2010 in the Paris prospective study 3 (PPS3), an observational prospective study that recruited 10,157 volunteers aged 50 to 75 years who were examined for free in a large preventive health centre between May 2008 and June 2012 in Paris (France).^14^ This preventive health centre is subsidized by the French national health care system and offers all working and retired individuals and their families a free medical examination. Subjects were selected and categorized as reported in Figure 1. From this study we excluded subjects with factors that can affect arterial properties and baroreflex function (overt cardiovascular disease, diabetes (treated or not), smoking, family history of premature cardiovascular diseases or antihypertensive and lipid-lowering drugs). This study has received institutional support by INSERM (N° C07–39) and is registered in the international trial registry (NCT00741728). The study protocol was approved by the Ethics Committee of the Cochin Hospital (Paris), and all participants provided written informed consent.

### Definitions of Risk Factors

Metabolic syndrome was defined according to the American Heart Association/National Heart Blood and Lung Institute criteria^17^ by the presence of 3 or more of the following components: (a) central obesity (waist circumference ≥94 cm in men and ≥80 cm in women); (b) hyperglycemia (glucose ≥100 mg/dL [5.6 mmol/L]); (c) low high-density lipoprotein (≤40 mg/dL [1.03 mmol/L] in men and ≤50 mg/dL [1.29 mmol/L] in women); (d) high triglycerides (≥150 mg/dL [1.7 mmol/L]); and (e) blood pressure ≥135/85 mm Hg.

### Protocol

All participants were studied in a quiet room with a controlled temperature of 22 ± 1°C and in steady state as previously described.^15^ In each subject, the arterial parameters and BP were measured at rest. BP was measured 3 times with an automated device using an oscillometric method (OMRON 705C); the mean of the last 2 measurements was used in this analysis. Consecutively, a complete noninvasive carotid artery study was performed.

### Ultrasound Method

Longitudinal B-mode (60 Hz, 128 radiofrequency lines) and fast B-mode (600 Hz, 14 radiofrequency lines) images of the right common carotid artery 2 cm below the carotid bulb were obtained using a high-precision echotracking device (ART.LAB^®^, Esaote, Maastricht, NL) paired with a high-resolution linear array transducer (7.5 MHz). One 6-second acquisition was done in the B-mode and the fast B-mode, and then 1 long 300-s recording with fast B-mode settings was performed.

### Carotid Parameters

The common carotid distension rate (Δ*d*/Δ*t*) was calculated as the rate of cyclic change in the distension of the arterial wall.^13^ Carotid stiffness was calculated from the time delay between 2 adjacent distension waveforms.^18^ R-R intervals were derived from the time difference between marks placed on the foot of the carotid diameter curve over the 5-min time period acquired at 600 Hz.

### Baroreflex

The concurrent beat-by-beat carotid distension rate and R-R interval were acquired for a minimum period of 300 s free of ectopic beats, arrhythmic events, missing data, and noise effects. A window of 256 heartbeats was selected for spectral analysis. The frequency contents of the variations in carotid artery diameter and the R–R interval signal were obtained by means of fast Fourier transformation using validated tools.^16^ The transfer function magnitude between output (R–R interval) and input (carotid distension rate) within the frequency band of 0.04 to 0.15 Hz defined the low-frequency gain and corresponded to the NBP.^19^

### Statistical Analysis

Statistical analyses were performed using the NCSS 2007 software (Gerry Hintze, Kaysville, UT). NBP is expressed as the median (interquartile range); all other data are expressed as the mean (standard deviation). Variables with a skewed distribution (ie, NBP) were log-transformed. Analysis of variance was used to compare different groups. Multiple comparisons were performed using the Bonferroni correction and a threshold of 0.017 (0.05/3) was used to assess statistical significance. Multivariate linear regression analysis was used to explore the correlation between NBP and different variables. *P* < 0.05 was accepted as statistically significant except for 2 by 2 comparisons.

## RESULTS

A flowchart describing how subjects were selected and then subdivided according to the presence of risk factors for MetS is presented in Figure 1, Panel A. The complete PPS3 database of patients enrolled from May 2008 to December 2010 contains data from 7026 subjects. BRS and relevant clinical data were available in 4705 subjects. Of those, 1870 subjects with overt cardiovascular disease, diabetes (treated or not), smoking, familiarity for premature cardiovascular diseases or antihypertensive, and lipid-lowering drugs were excluded according to the study design. A total of 2835 subjects met the selection criteria (Figure 1, Panel A) and were included in this report (701 subjects with 0 components for MetS, hereafter reported as “controls,” 1673 subjects with 1 to 2 components for MetS, hereafter reported as “subjects at risk for MetS,” and 461 subjects with 3 to 5 components for MetS, hereafter reported as “MetS subjects”).

### Neural Baroreflex Pathway, Carotid Stiffness, and Metabolic Syndrome Components

**FIGURE 2:**
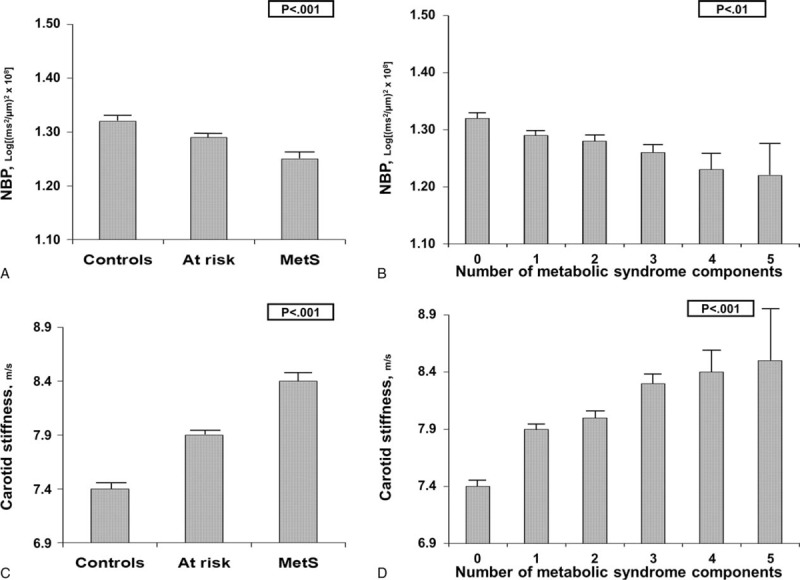
Neural baroreflex pathway (NBP) and carotid stiffness in controls, subjects at low-risk and subjects with metabolic syndrome (MetS): mean values and standard error of the mean according to MetS categories (panel A and C) or number of MetS components (panel B and D). MetS = metabolic syndrome, NBP = neural baroreflex pathway.

**FIGURE 3:**
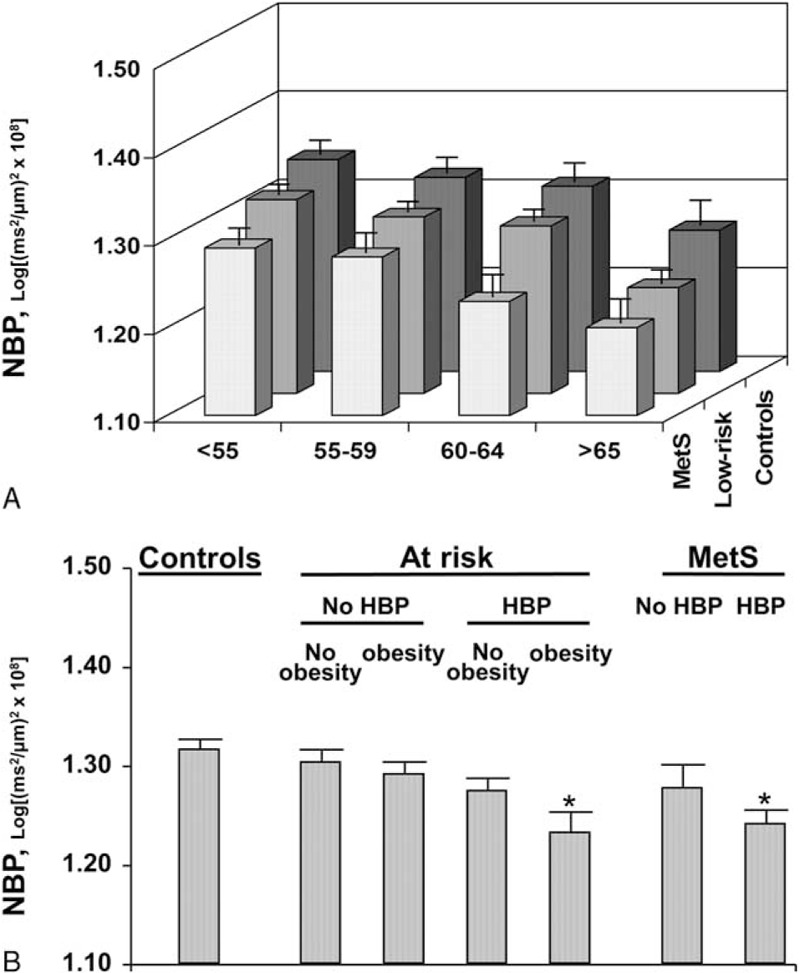
Mean values and standard error of the mean of neural baroreflex pathway (NBP) in controls, subjects at risk for metabolic syndrome (MetS) and subjects with MetS. Panel A: stratification for age categories. Panel B: Stratification for blood pressure ≥135/85 mm Hg (HBP) and obesity. ^∗^significantly different from controls (univariate ANOVA with Bonferroni corrections). MetS = metabolic syndrome, NBP = neural baroreflex pathway

The prevalence of MetS was 20.1% in men and 10.4% in women. Compared with controls, subjects with MetS and those at risk for MetS had lower NBP (−5.3% and −2.3%, respectively; linear trend, *P* < 0.001) and higher carotid stiffness (+13.5% and +6.8%, respectively; linear trend, *P* < 0.001) (Table 1 and Figure 2, Panel A and C). A reduction of NBP in subjects with an increasing number of MetS components was reported in each age group and was more evident in subjects >65 years (Figure 3, Panel A). NBP was significantly reduced in subjects at risk for MetS (*P* = 0.001) and in those with MetS (*P* < 0.001) even after correction for age, sex, and carotid stiffness (Table 2, Model A). Further analysis revealed that obesity and high blood pressure were the major factors associated with reduced NBP in subjects with MetS. The separate influence of the 5 components of metabolic syndrome on NBP, adjusted for age, sex, and carotid stiffness, is reported in Table 2—Model C.

**TABLE 1:**
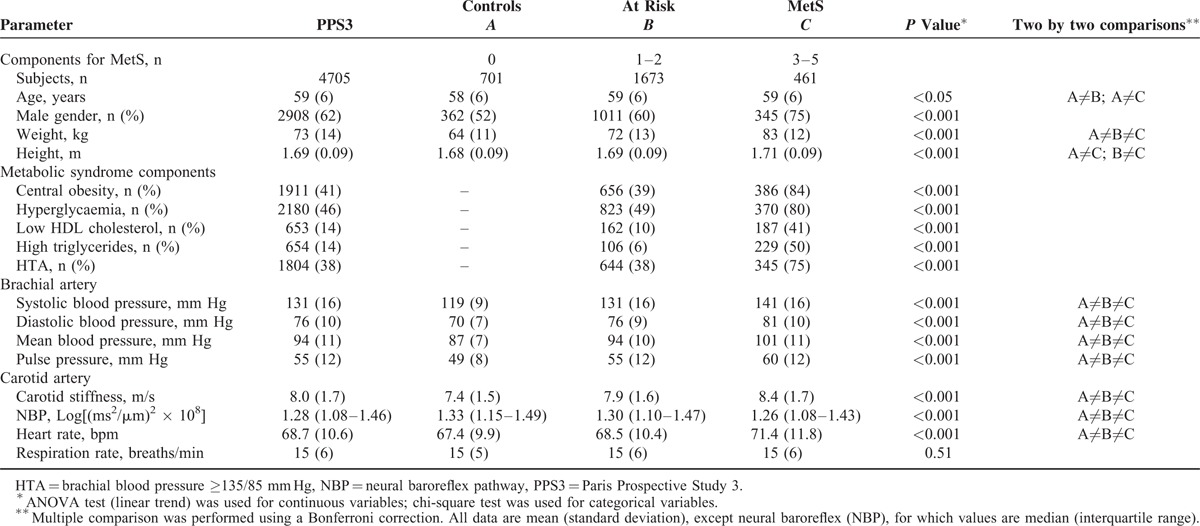
Description of General Clinical Parameters of Subjects With Different Number of Components for Metabolic Syndrome

**TABLE 2:**
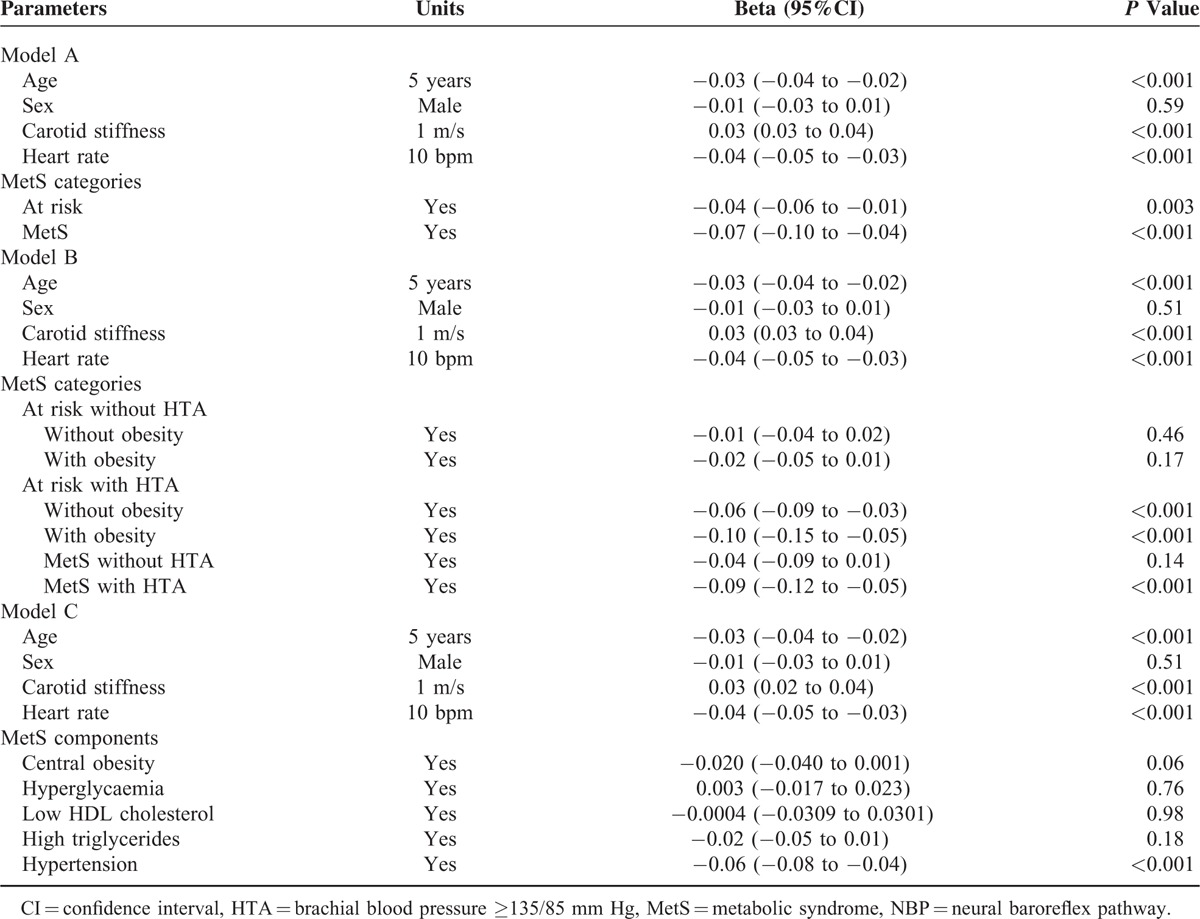
Multivariate Analysis for Neural Baroreflex Pathway (NBP)

### Blood Pressure, Obesity, and the Neural Baroreflex Pathway

The role of BP and obesity on the reduction of NBP in subjects with an increasing number of MetS components was tested. Subjects at risk for MetS and those with MetS were categorized according to the presence of brachial BP ≥135/85 mm Hg. Subjects at risk for MetS, with and without brachial BP ≥135/85 mm Hg, were subsequently categorized according to the presence of obesity. The reduction of NBP in subjects at risk for MetS and in those with MetS was confirmed only in the subgroup with brachial BP ≥135/85 mm Hg regardless of obesity (*P* < 0.001; Table 2, Model B). In subjects with brachial BP <135/85 mm Hg, NBP did not differ between controls, subjects at risk for MetS (regardless of obesity) and subjects with MetS (Table 2, Model B, Figure 3).

### Neural Baroreflex Pathway and Carotid Stiffness

**FIGURE 4:**
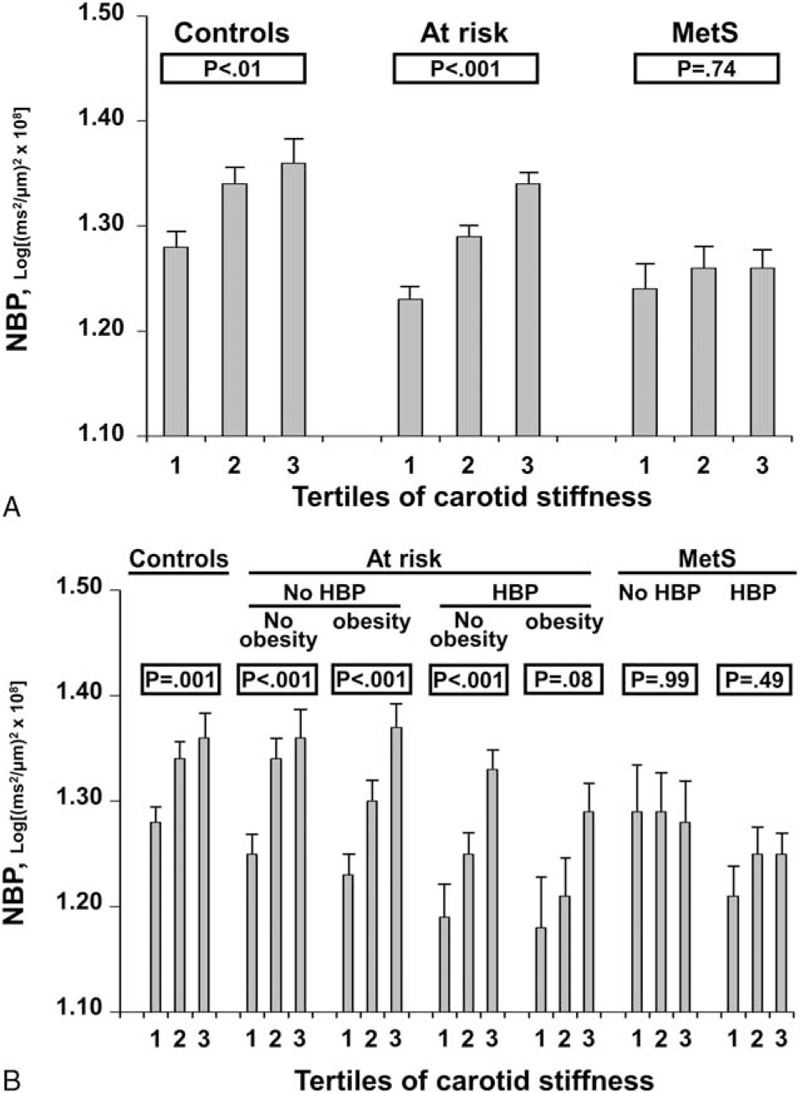
Neural baroreflex pathway (NBP) in controls, subjects at risk for metabolic syndrome (MetS) and subjects with MetS. Panel A: mean values and standard error of the mean according to tertiles of carotid stiffness. Panel B: mean values and standard error of the mean according to tertiles of carotid stiffness and presence of blood pressure ≥135/85 mm Hg (HBP) and obesity. MetS = metabolic syndrome, NBP = neural baroreflex pathway.

Controls, subjects at risk for MetS and subjects with MetS were categorized according to carotid stiffness tertiles (<7.1 m/s; 7.1–8.3 m/s; >8.3 m/s; Figure 4, Panel A). NBP increased from the lower to the higher tertile of carotid stiffness in controls (*P* < 0.01) and subjects at risk for MetS (*P* < 0.001). Inversely, the increase of NBP according to carotid stiffness tertiles was abolished in patients with MetS, in which NBP was comparable in each carotid stiffness tertile, regardless of the blood pressure status (Figure 4, Panel B).

## DISCUSSION AND CONCLUSIONS

For the first time, the present study assessed spontaneous NBP in patients with different numbers of MetS components using a new noninvasive technique, the cross-spectral analysis of carotid distension rate and heart rate. The major findings are reported as follows: (a) a progressive reduction of NBP was reported in patients with an increasing number of components of the MetS. (b) This reduction was largely associated with the presence of high BP. (c) We reported an increase of NBP with carotid stiffening in control subjects and in patients with 1or 2 components of the MetS. (d) The increase of NBP with carotid stiffening was depressed in subjects with MetS independent of the BP levels.

### Interpretation of Findings

In the present work, we reported that NBP is reduced in patients with MetS. To our knowledge, no previous studies have been carried out to evaluate the relationship between NBP and MetS. However, other studies have analyzed the association between *global* BRS, which is influenced by the vascular component of the baroreflex and NBP (Figure 1, Panel B) and MetS,^8^ or by the components of the syndrome separately.^20–23^ It is known that *global* BRS is reduced in hypertension,^20^ obesity,^21^ dyslipidaemia,^22^ and diabetes.^23^ In addition, it has been suggested that hypertension acts synergistically with type 2 diabetes to depress *global* BRS and that insulin resistance plays an important role.^24^ Finally, Grassi et al reported that the association between hypertension and obesity triggers a sympathetic activation and baroreflex impairment that is more extensive than that found in either of these conditions considered separately.^25^ Taken together, these studies indicate that *global* BRS is impaired in the presence of the separate components of MetS, as well as in subjects with MetS. In the present work, we extend the results of previously published papers, suggesting that the neural component of the baroreflex is altered, even after fully controlling for the vascular component.

Baroreceptors respond to deformation and not to pressure per se.^9^ Therefore, changes in peripheral BP might not accurately reflect changes in carotid bulb distension, particularly in patients with carotid stiffening. The vascular component of the baroreflex and NBP may be altered in many pathological conditions associated with MetS. Greater carotid vascular stiffness depresses the autonomic regulation of the baroreflex in hypertensive patients.^10^ Therefore, considering that BP abnormality represents 1 of the 5 components that lead to the identification of MetS, it is unsurprising that the vascular component (ie, aortic and carotid stiffness) may be increased in these subjects^11^ and that the *global* BRS may not accurately reflect the NBP. Alternatively, in the absence of structural changes, autonomic dysfunction reduces neural transduction and dampens the responses of the baroreflex to decreases in BP in diabetic patients.^12^ Considering that autonomic dysfunction may begin with alterations of the vascular component, whether the alteration of the *global* BRS in subjects with MetS is fully explicable with the alteration of the vascular component alone or with alterations of both the vascular component and NBP is unknown. In the present work, we derived NBP from fluctuations of carotid distension,^13^ thereby allowing the study of the neural path of the baroreflex after fully controlling for the vascular component. We reported that the NBP is altered in patients with MetS independent from the alteration of the vascular component of the baroreflex (ie, elevated carotid stiffness). In addition, the observation that the impairment of NBP was inversely related to the number of the various metabolic components of the syndrome (Figure 2) suggests a cumulative effect of MetS components on autonomic dysfunction.

Another important finding of the present report is that the reduction of NBP reported in patients at risk for MetS and in those with MetS is BP-dependent (Figure 3, Panel B). This finding confirms the strong association between high BP and autonomic dysfunction.^20,24^

Although originally described many decades ago,^26^ much debate has continued regarding the recognition of MetS as a real syndrome and whether it is an informative clinical tool. Some authors claimed that MetS is not a single pathophysiological entity, that its identification has neither pedagogical nor clinical utility, and that clinical emphasis should rather be placed on effectively treating any cardiovascular risk factor that is truly present.^27,28^ However, the current opinion is that MetS confers an increased risk of cardiovascular events, which is attributable only in part to the individual risk factors that concur in defining it.^29^ In the present work, we reported that the increase of NBP according to carotid stiffness tertiles is detectable in controls and subjects at risk for MetS but is lost in subjects with MetS (Figure 4, Panel A). Interestingly, when we attempted to dissect the relative contribution of BP in determining the lack of increase of NBP in MetS with carotid stiffening, we found that the autonomic abnormalities were manifest, even when hypertensive patients were excluded (Figure 4, Panel B). Taken together, these findings suggest that the NBP may be stimulated by an increase of carotid stiffness in both normotensive and hypertensive subjects in the absence of MetS and specifically depressed by MetS as a whole. Our data are consistent with the hypothesis that the dysfunction of the baroreflex is a characteristic of the syndrome independent of BP elevation and represents an intrinsic feature of this clinical condition.^8,30^

The study of autonomic function in patients with MetS is crucial for both prognosis and therapeutic strategy. Interestingly, from the different tests used to evaluate autonomic function, spectral analysis is able to identify an impairment of the baroreflex's control of the heart rate at a time when traditional testing still yields normal results,^23^ suggesting the superiority of this technique over traditional laboratory procedures in the early detection of autonomic abnormalities.^31^ Therefore, the implementation of the noninvasive assessment of spontaneous NBP in the routine evaluation of patients with MetS could help to select those at high cardiovascular risk, which can lead to improvements in autonomic function and, consequently, the patient's prognosis.

However, looking at the results of the present work, many questions remain to be clarified. The prognostic relevance of the lack of an increase in NBP in subjects with carotid stiffening remains to be determined not only in patients with metabolic syndrome but also in the general population. Moreover, considering that the impairment in cardiac autonomic regulation could precede the onset of hypertension,^32^ it could be interesting to prospectively study whether the lack of NBP increase in subjects with carotid stiffening is able to predict the onset and the progression of hypertension in subjects with normal BP. These questions will be addressed at the end of the on-going 10-year longitudinal follow-up of the large cohort of the PPS3 study.

### Methodological Features and Limitations of the Study

The present study has several strengths. First, we used recognized methods for measuring arterial parameters:^15^ an echotracking apparatus (ART.LAB® system), coupled with one of the most powerful noninvasive techniques used to measure the baroreflex,^19^ all integrated with cross-spectral analysis and validated tools^16^ in semiautomated processing. Second, we measured the NBP using the carotid distension rate instead of the peripheral BP,^13^ allowing the study of the neural path of the baroreflex after fully controlling for the vascular component. Third, we performed this analysis in a large epidemiological study.^14^

The present study has limitations. First, the cross-sectional design may limit the ability to infer a causal relationship. Second, the technique applied was indirect. Third, the data acquisition required particular skill, and the analysis remained complex. Fourth, we assessed only 1 aspect of baroreflex regulation, the baroreflex heart rate regulation, but not the baroreflex regulation of vascular tone. Fifth, traditional baroreflex measurements based on beat-by-beat blood pressure were not available. Finally, insulin resistance was not measured.

In conclusion, we observed that NBP is impaired in subjects with MetS. This impairment is only partially the consequence of BP abnormalities, as it is also influenced by altered carotid stiffness. Finally, we reported an increase of NBP in subjects with carotid stiffening in controls and subjects with 1 to 2 components of MetS but not in those with MetS.
